# Machine learning in time-lapse imaging to differentiate embryos from young vs old mice^†^

**DOI:** 10.1093/biolre/ioae056

**Published:** 2024-04-30

**Authors:** Liubin Yang, Carolina Leynes, Ashley Pawelka, Isabel Lorenzo, Andrew Chou, Brendan Lee, Jason D Heaney

**Affiliations:** Division of Reproductive Endocrinology and Infertility, Department of Obstetrics and Gynecology, Baylor College of Medicine, Houston, Texas, USA; Division of Reproductive Endocrinology and Infertility, Division of Reproductive Sciences, Department of Obstetrics, Gynecology, and Reproductive Sciences, Yale School of Medicine, New Haven, Connecticut, USA; Department of Molecular and Human Genetics, Baylor College of Medicine, Houston, Texas, USA; Department of Molecular and Human Genetics, Baylor College of Medicine, Houston, Texas, USA; Department of Molecular and Human Genetics, Baylor College of Medicine, Houston, Texas, USA; Department of Molecular and Human Genetics, Baylor College of Medicine, Houston, Texas, USA; Pain Research, Informatics, Multi-morbidities, and Education (PRIME) Center, VA Connecticut Healthcare System, West Haven, Connecticut, USA; Section of Infectious Diseases, Department of Internal Medicine, Yale School of Medicine, New Haven, Connecticut, USA; Department of Molecular and Human Genetics, Baylor College of Medicine, Houston, Texas, USA; Department of Molecular and Human Genetics, Baylor College of Medicine, Houston, Texas, USA

**Keywords:** machine learning, morphokinetics, preimplantation mouse embryos, time-lapse microscopy, maternal aging, predictive modeling

## Abstract

Time-lapse microscopy for embryos is a non-invasive technology used to characterize early embryo development. This study employs time-lapse microscopy and machine learning to elucidate changes in embryonic growth kinetics with maternal aging. We analyzed morphokinetic parameters of embryos from young and aged C57BL6/NJ mice via continuous imaging. Our findings show that aged embryos accelerated through cleavage stages (from 5-cells) to morula compared to younger counterparts, with no significant differences observed in later stages of blastulation. Unsupervised machine learning identified two distinct clusters comprising of embryos from aged or young donors. Moreover, in supervised learning, the extreme gradient boosting algorithm successfully predicted the age-related phenotype with 0.78 accuracy, 0.81 precision, and 0.83 recall following hyperparameter tuning. These results highlight two main scientific insights: maternal aging affects embryonic development pace, and artificial intelligence can differentiate between embryos from aged and young maternal mice by a non-invasive approach. Thus, machine learning can be used to identify morphokinetics phenotypes for further studies. This study has potential for future applications in selecting human embryos for embryo transfer, without or in complement with preimplantation genetic testing.

## Teaser

Maternal aging in murine in vitro fertilization shows faster cleavage and compaction stages of embryonic development by time-lapse microscopy, and artificial intelligence predicts the aging phenotype.

## Graphical Abstract

**Graphical Abstract:**
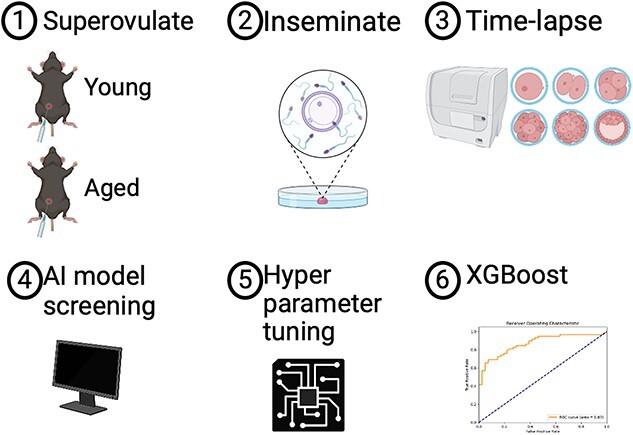

## Introduction

Over the past decade, time-lapse microscopy with concurrent embryo culture has emerged as a revolutionary non-invasive technology in the field of human reproduction. This innovation enables continuous embryo culture while capturing multi-planar images every 10–30 min [1, 2]. These images record the stages of preimplantation embryonic development, from fading of the pronuclei to blastulation. The process of tracking the temporal events during embryogenesis is termed embryo morphokinetics.

Morphokinetics has significant applications in human in vitro fertilization (IVF) clinics [1, 2], particularly in selecting viable embryos for implantation, possibly supplementing preimplantation genetic testing and improving pregnancy [3–6]. Changes in embryo morphokinetics are linked to many phenotypes including blastocyst formation [7–10], aneuploidy status [11–18], ovarian disease [19–21], and pregnancy rates [4, 22–34].

Morphokinetics also serves as a critical tool in mouse preimplantation embryo research. The mouse presents as a robust model for in-depth embryological studies due to its extensive genetic and morphokinetics studies [35–40]. However, there is currently a knowledge gap in understanding the detailed molecular events that govern the preimplantation embryo morphologic development [41, 42]. Recent studies show correlations between biomarkers and embryo morphokinetics [43–48]. Further studies in non-invasive embryo morphokinetics would allow researchers to couple invasive molecular tests such as RNA-sequencing for gene signatures with morphokinetic patterns. One method to improve the study of morphokinetics is to add machine learning as in human studies noted above.

Machine learning is particularly useful for analyzing, characterizing, and automating the analysis of large datasets (many embryos) with complex features (multiple time points or images), both of which are present in morphokinetics [49]. There are two types of machine learning, supervised and unsupervised. Supervised learning refers to running different mathematical models that best fit the data to predict an outcome. Here, the investigator labels the predictor variables, such as morphokinetic time points, and uses one or multiple algorithms with different parameter settings to best predict the outcome variable (such as young vs aged). Many mathematical models can be used including regression, classification models [49]. In contrast, unsupervised learning refers to the use of mathematical models to find patterns in the data (such as slow or fast morphokinetics) without any prior knowledge of the type of data (whether embryos came from aged or young mice). Unsupervised algorithms include clustering methods such as principle component analysis [49]. A third type of newer machine learning that requires large computational power is deep learning, or artificial neural networks. Deep learning is useful to analyze very large datasets such as the pixels from embryo time-lapse images [50, 51]. With recent advancements in computational power, numerous studies now employ artificial intelligence (AI) and deep learning technologies [50, 52–54] to analyze still images [55–58] and videos [59] of embryo development.

In this study, we investigated the impacts of maternal aging on preimplantation development using morphokinetics. Studies of embryos from women of advanced reproductive age show significant changes in individual morphokinetics time points [60] that are likely related to poor response to hormonal stimulation [61]. The mouse has been used as a model of ovarian aging as aged maternal mice have decreased oocytes, increased aneuploidy rates, and decreased litter sizes [62–64]. By comparing embryos derived from both young and aged oocytes, we aim to uncover potential morphokinetic phenotypes associated with maternal aging using machine learning.

## Materials and methods

### Generation of embryos

All animal work was conducted in accordance with the Institutional Animal Care and Use Committee standards and guidelines at Baylor College of Medicine. All necessary ethical standards for conducting research involving animals have been rigorously followed. All mice were housed in a temperature-controlled environment with 12 h light and dark cycles at the B4 barrier level. Both young (3–4 weeks old) and aged (10–14 months old) female C57Bl6/NJ mice underwent ovarian hyperstimulation. Superovulation was induced by intraperitoneal injection of 5 IU pregnant mare serum gonadotropins during random cycle followed by 5 IU of human chorionic gonadotropin 48 h later. Oocytes were harvested 16–18 h later as previously described [65–67]. Briefly, a laparotomy incision was performed followed by oviduct excision under dissection microscopy. An incision was made on the oviduct and cumulus oocyte complexes were harvested in human tubal fluid (HTF) media (Millipore). The oocytes were inseminated by conventional method with fresh sperm from a 4–5 month-old male C57Bl6/NJ donor [65–67]. Briefly, sperm was harvest from the cauda epididymis, incubated in methyl-b-cyclodextrin with modified Krebs–Ringer bicarb solution (THY). Zygotes were washed, sorted, and manually counted in HTF media (Millipore).

### Embryo culture

Fertilized zygotes were isolated and cultured in 20 uL of potassium simplex optimization medium (KSOM) (Millipore) in Embryoscope Plus (Vitrolife) incubator under humidified atmosphere at 37 °C with 6% CO_2_ and 20% atmospheric oxygen. Embryos were observed under time-lapse microscopy until embryonic Day 4.5. Images were obtained every 12 min at 11 focal planes under bright field microscopy. Multiple experimental cohorts were conducted to minimize bias (replicate group 1 *n* = 80, replicate group 2 *n* = 133, replicate group 3 *n* = 143, replicate group 4 *n* = 47). Details of the number of aged, young, and arrested embryos for each group are shown in Supplementary Figure 1A. Each experimental cohort contained at least five maternal donors. For each experiment, sperm from one 4–5-month donor C57/Bl6/NJ male mouse was used to inseminate all oocytes. Each maternal donor contributed approximately 4–10 oocytes as oocytes were pooled, washed, inseminated, and then counted. Each maternal donor contributed 3–23 embryos (young group) and 4–21 embryos (aged group).

### Morphokinetics annotations

Following time-lapse microscopy, embryo images were exported from the EmbryoViewer software (Vitrolife) at the plane with the best focus of the image. Images were manually reviewed until 94.5 h post-insemination. The time at which embryos reached a developmental milestone was recorded. Each embryo received a code that blinded the phenotype from the person performing annotations. The person annotating was trained by an embryologist and IVF lab director at the Texas Children’s Family Fertility Center. Fourteen morphokinetics time points were manually annotated for each embryo from time to fading of pronucleus to time to expanded blastocyst. Time points were defined as follows: tPNf—time to fading of the pronuclei, t2—time to reach 2-cell stage, t3—3-cell, t4—4-cell, t5—5-cell, t6—6-cell, t7—7-cell, t8—8-cell, t9—9-cell, tM—morula (>80% fading of cell membrane/compaction), tSB—development of small pocket of blastocele, tB—development of 50% blastocele, tEB—development of enlarged blastocyst with thinned zona pellucida.

### Statistical analysis

A statistical consult was made with the institutional statistics team (Biostatistics and Analytics Group of the Biostatistics and Informatics Shared Resource). Analysis methods were recommended by the statistics team. Analysis was performed by authors. Two-way analysis of variance (ANOVA) was used to compare differences among all the time points. Kaplan–Meier survival estimates were used to compare the two experimental cohorts for each time-to-event (time point). Time in hours was a continuous variable. The log-rank test for equality of survivor functions was used to compare differences in time-to-event between the two cohorts. Statistical analysis and visualizations were performed with JMP Pro, Prism, and Stata.

### Unsupervised machine learning

Embryos that arrested prior to reaching blastocyst stage were excluded. Data from all experimental cohorts were pooled. The Normal Mixtures model was used for unsupervised clustering due to overlapping distributions and its specificity for numerical data. The means, standard deviations, mixture proportions, and correlations were calculated from the maximum likelihood estimation (expectation–maximization [EM] algorithm) [68]. Number of clusters tested ranged from 2 to 12 using JMP Pro 17. Default parameters used included 30 tours, 300 maximum iterations, and 1e-8 converge criterion.

### Supervised machine learning

Embryos that arrested prior to reaching blastocyst stage were excluded from machine learning analysis. Morphokinetic data of embryos that reached the blastocyst stage from all experimental cohorts were uploaded to Jupyter Notebook 6.5.4 through Anaconda Navigator [69]. Predictors were all morphokinetics time points in hours. Outcome variable Age was encoded as a binary variable of 0 = Young and 1 = Aged. Total data were split into 70% training set and 30% test. Predictive models tested were XGBoost (extreme gradient boosting) [70], random forest [71], and logistic regression [72] using scikitlearn [73]. Further hyperparameter tuning was conducted on the test data with five-fold cross validation (splitting data into five sections and training four sections and validating on one section with repeats until all sections are tested). Hyperparameters tuned for logistic regression were: penalty of l1, l2, elasticnet, or none; C of np.logspace −4, 4, and 20; solver of lbfgs, newton-cg, liblinear, sag, and saga, max_iterations. For random forest classifier: n_estimators of 200 and 500, max_features of auto, sqrt, anog2; max_depth of 4 to 8; criterion of gini and entropy. For XGBoost: min_child_weight 1, 5, 10; gamma 0.5 to 5; subsample 0.6 to 1.0; colsample_bytree 0.6 to 1.0, max_depth 3 to 5. Confusion matrices were generated.

## Results

### Morphokinetic study

**Figure 1:**
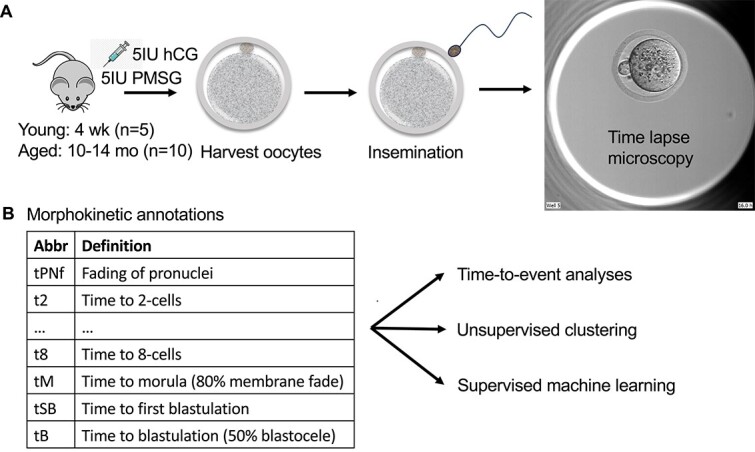
Schematic of methods. (A) Two cohorts of young (*n* = 5) and naturally aged (*n* = 10) female mice underwent ovarian hyperstimulation, oocyte harvest, and in vitro insemination, with time-lapse microscopy. (B) Following incubation of zygotes to the blastocyst stage, still images of each embryo were annotated manually and analyzed using statistical methods in the figure.

We sought to determine the morphokinetic parameters of in vitro embryo growth comparing young versus aged oocyte donors. To do this, we superovulated young (4 week-old) and aged (10–14 month-old) female C57BL6/NJ mice by injecting pregnant mare serum gonadotropins (PMSG) followed by human chorionic gonadotropin (hCG) (Figure 1A). Oocytes from both cohorts were harvested from the oviduct and were inseminated with fresh sperm from a 4–5 month-old male C57BL6/NJ mouse. Following insemination, the zygotes were placed under time-lapse microscopy in the Embryoscope Plus incubator for 95 h post-insemination. Images during the time-lapse were reviewed, and each embryonic developmental hour was annotated manually (Figure 1B). Each time point was collected and compared for each embryo between the aged and young cohorts using time-to-event and machine learning methods.

### Comparisons between young and aged at each time point

**Figure 2:**
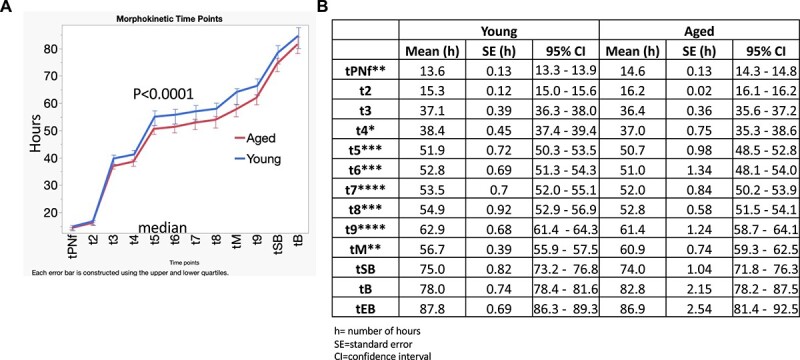
(A) Comparison of overall difference in the median number of hours for each morphokinetic time point between young (*n* = 15) and aged (*n* = 65) cohorts of embryos using two-way ANOVA (mixed-effect analysis of time vs age). Error bars represent upper and lower quartiles. (B) Further comparison of individual time points for each embryo using Kaplan–Meier survival estimates in the same experimental group. ns = non-significant, ^*^P ≤ 0.05, ^*^^*^P ≤ 0.01, ^*^^*^^*^P ≤ 0.001, ^*^^*^^*^^*^P ≤ 0.0001, 0 = young, and 1 = aged embryos.

The number of mice and embryos per experimental group are listed in a table in Supplementary Figure 1A. There was no difference in the total number of embryos obtained for each group for each experimental cohort. More mice were used in the aged group to compensate for fewer number of oocytes. Fewer oocytes are obtained after superovulation with aged female mice [64] and thus leading to fewer number of embryos. Recently, human embryo studies show that embryos derived from aged mothers were more prone to arrest at four to seven cell stages though the morphokinetics timings did not reach statistical significance difference [74]. To test the effect of maternal aging on murine embryo development, we compared the proportion of embryos that reached each developmental stage. We did not find a difference in the number of embryos that arrested between the two groups (Supplementary Figure 1B). To determine whether there were differences in embryo development between the aged (*n* = 8–13 mice) and the young (*n* = 5 mice) cohort of embryos, first, we used two-way ANOVA and found a significant difference between the two cohorts (Figure 2A).

To compare each time point in this experiment, we used time-to-event analysis in which, for instance, tPNf (time to pronucleus fade) or t8, was treated as a time-to-event variable. We found that the embryos derived from the aged mothers (*n* = 65) had significantly faster or shorter time to reach cleavage stages of 5-cells, 6-cells, 7, and 8-cells and compaction also known as morula formation (tM) than the young (*n* = 15) (Figure 2B for each time point). The comparisons of the mean time points between young and aged embryos are shown in Figure 2B. This experiment was replicated three times (the number of embryos per experiment and time-to-event analysis of each time point for the first experiment are shown in Supplementary File 1). The results demonstrate that the embryos derived from aged maternal donors were faster to reach the cleavage stages of 5- to 8-cells and compaction but were not faster to blastulate.

### Unsupervised clustering of blastocysts

**Figure 3:**
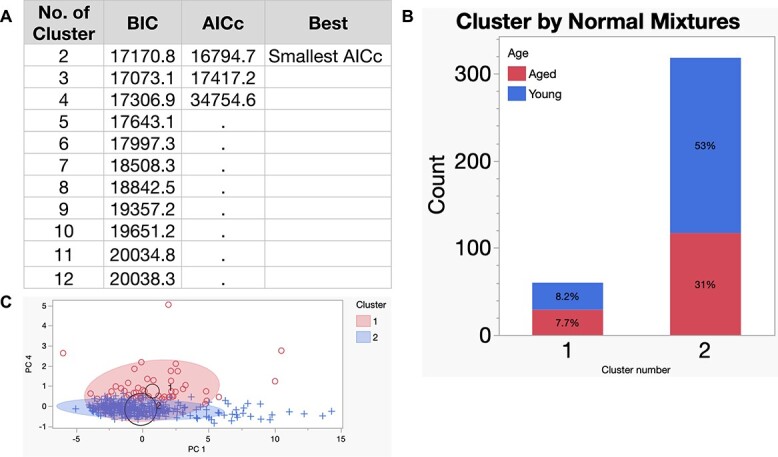
Unsupervised clustering by Normal Mixtures. (A) Comparison of cluster performance by corrected AICc and the BIC. (B) Number of embryos in each cluster by normal mixtures clustering method. Normal mixtures model was used for unsupervised clustering due to overlapping distributions and its specificity for numerical data. Percent of aged or young embryos in each cluster. (C) Visual representation (biplot) of distribution of data for each cluster by PCs 1 and 4. Shaded area represents contour density with 90% confidence interval. Top oval with circular dots represents cluster 1 and thin oval with + signs represents cluster 2. Black circles surround the cluster center and their sizes are proportional to the count for each cluster.

Next, we asked whether machine learning techniques could distinguish between the two cohort of blastocyst embryos using an unsupervised clustering approach. Missing data for each time point represented embryos that arrested prior to reaching blastocyst stage. These embryos were thus excluded as they likely represented a different biological groups of aged embryos. Here, we used the Normal Mixtures method to cluster the remaining embryos (*n* = 328) based on the number of hours for each time point without any scientific input beforehand (i.e., the algorithm did not know a priori which embryos were from the aged (*n* = 146 or young (*n* = 182) cohorts). To determine the optimal number of clusters, we reran this algorithm with 2, 3, etc., and up to 12 clusters. We compared the performance of each clustering analysis by assessing their Bayesian Information Criterion (BIC) and Akaike Information Criterion (AICc) scores (Figure 3A). The number of clusters with the best performance was 2. To determine the distribution of aged and young embryos in each cluster, we graphed the proportions of young and aged embryos in each cluster (Figure 3B). Cluster 2 had higher count and percentage of embryos in the aged cohort (63% vs 37%), while Cluster 1 appeared to have equal representation of aged (48%) and young (52%) embryos (Figure 3B). Spatial visualization of the two clusters by principal components (PC) showed that there was overlap between the two clusters (Figure 3C). Therefore, these data showed that embryo morphokinetic patterns can be clustered into two primary clusters with one predominantly containing young embryos.

### Supervised machine learning

**Figure 4:**
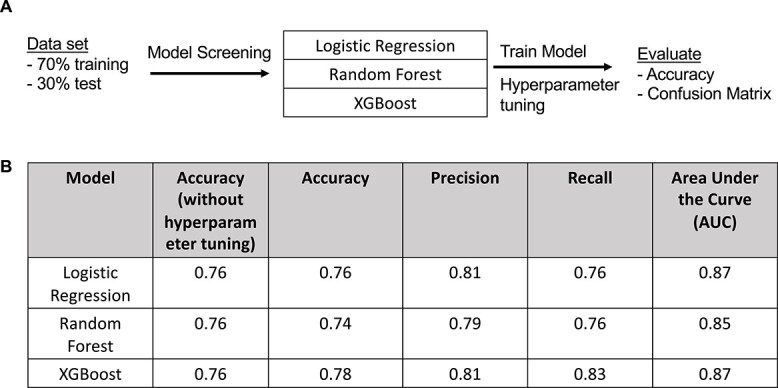
(A) Diagram of supervised machine learning strategy and (B) performance of each model before and after hyperparameter tuning on the test set.

We then asked whether embryo morphokinetics can predict phenotype using non-invasive time lapse microscopy. To do this, we used supervised machine learning algorithms to predict whether the embryo was from the aged or young cohorts. We split the data into 70% training set and 30% test set and trained on three models of logistic regression, random forest, and XGBoost (Figure 4A). The original dataset is shown in Supplementary File 2. Each model gave accuracy of 0.76. Therefore, each model underwent hyperparameter tuning with 5-fold cross validation to prevent overfitting, totaling 2025 model fits. After tuning, the model that performed the best was XGBoost (Figure 4B) with the following hyperparameters:

colsample_bytree = 0.8, gamma = 2, max_depth = 3, min_child_weight = 1, subsample = 0.8

The XGBoost model performed best of the three with an accuracy of 0.78, precision 0.81, and recall 0.83. Confusion matrices are shown in Supplementary File 1. For the XGBoost algorithm, 20 embryos were misclassified from total of 114 in the test set. Out of the 20, 9 were misclassified as “aged” and 11 were misclassified as “young.” Therefore, machine learning models with hyperparameter tuning can predict the aged phenotype of mouse embryos in this dataset.

## Discussion

These findings provide several scientific insights. First, the results demonstrate that embryos from aged mothers had significantly faster cleavage stages and compaction and relatively unchanged blastulation compared to that of the young group. This implies that despite having faster development at cleavage and compaction stages, the embryos from aged mothers had relatively slower blastulation that matched that of the young cohort. Similarly, in human embryo data, maternal age significantly impacts the morphokinetics [60] and is associated with not only faster cleavage stages [75] but also slower blastulation development [76, 77]. In human IVF, slower blastulation is associated with lower pregnancy and higher aneuploidy rates [78]. Faster cleavage is also seen in other diseases such as endometriosis [79], maternal obesity [80], and polycystic ovarian syndrome [19]. Indeed, more studies are needed to understand the impact of aging on preimplantation embryo growth [81, 82].

Interestingly, abnormal cleavage rates such as delayed cleavage of zygotes are associated with abnormal chromosomal segregation, caused by the activation of the spindle assembly checkpoint (SAC) [83]. During cell division, the SAC safeguards against chromosomal errors by halting the cell cycle progression until chromosomes are properly aligned [84]. In aging, the SAC becomes activated likely due to DNA damage accumulated in embryos from aged oocytes. The SAC may explain the change in the morphokinetics found in this study. Further analysis is needed on the level and role of the SAC on embryo development in maternal aging.

This study shows that the embryos from aged maternal donors were slower to complete fading of the pronuclei (tPNf). The breakdown of the pronuclei on microscopy represent integration of the male and female chromosomes. Similarly, in human embryo, delayed fading of the pronuclei is associated with poor oocyte quality [85], maternal aging [77], and decreased embryo viability [86]. Maternal aging is also linked with greater oxidative stress, abnormal gene expression, DNA damage, chromosomal segregation errors, and abnormal stringency of the SAC [87–89]. Thus, we show that aged maternal oocytes induce delayed pronuclear breakdown likely attributable to abnormal oocyte quality.

Notably, there was no difference in the proportion of embryos that underwent developmental arrest in the aged maternal donors, though there was a trend for human embryos that arrest on Days 4–7 in other studies [74]. This could be explained by either a needing a greater number of embryos to detect a difference or that perhaps there are intrinsic differences between the murine and human embryos.

In this study, cleavage stages and morula formation were altered in embryos from aged mothers. Development of the cleavage stages coincides with embryonic genome activation and detection of paternal transcripts in humans [90–92]. The cleavage stages in the human embryo correlate with blastulation, ploidy status, and implantation rate [10, 93, 94]. The activation of the zygotic genome may potentially be altered in the aged embryos.

Interestingly, our study also highlights the alteration of the morula and blastocyst stages in maternal aging. There could be several reasons for this observation. First, delayed blastulation (time from fading of pronuclei to blastocyst stage) occurs in embryos with multi-pronuclei (PN) in bovine models [95] and is associated with abnormal fertilization [96]. Multi-PNs in human zygotes are also associated with high rates of aneuploidy [97, 98]. Multi-PN zygotes were not excluded from this study, and thus, the presence of multi-PN could have explained why the aged embryos had delayed blastulation. For this study, we were unable to determine the number of multi-PN zygotes. The mouse strain used in this study, C57/BL6, is known to have granular cytoplasm and small pronuclei, making it difficult to visualize the total number of pronuclei compared to other strains such as Friend leukemia virus B (FVB) that have clear cytoplasm and large pronuclei [99]. Therefore, a caveat to interpreting the data is that we did not account for the presence of multi-PN and thus, multi-PN could have explained the differences in phenotype. To characterize the effect of aging on the frequency of multi-PN, further studies either with the FVB mouse strain or with a novel pronuclear imaging techniques for the C57 strain would be needed. A second explanation of delayed compaction and blastulation is that these two stages represent many critical development changes. The blastocyst stage coincides with the completion of zygotic genome activation, lineage-associated gene expression heralding lineage commitment [92]. The morula and blastocyst formation also coincide with genome-wide nadir of DNA methylation as a landmark for stem cell pluripotency [100]. The morula stage is also concurrent with embryonic expression of E-cadherin and other adhesion molecules to establish cell-to-cell contact for compaction, required for cellular organization [41, 101]. At this time, two differentiated cell lineages develop within the morula: outer cells that become the trophoblast and inner cells that become the inner cell mass of the blastocyst [102]. Major cell reorganization, changes in symmetry, and suppression of many pluripotency genes such as *OCT4* along with upregulation of maternal effect epigenetic regulators such as *Kdm4a* take place as the morula transforms into a blastocyst [42, 103, 104]. These carefully coordinated events may be dysregulated in aged embryos that progress faster to the morula but slower to the blastocyst stage.

Second, the findings in this study demonstrate that AI algorithms can distinguish between embryos from aged and young maternal donors with as few as about 300 samples. Typically, greater sample sizes are needed for direct analysis of images with deep neural network models. This is because the model will need to be trained to recognize not only cells but also stages of development and how to classify normal from abnormal stages [105]. Here, the information in the images was condensed into morphokinetics continuous variables. Then, these variables can train predictive machine learning models with modern processors with as few as 5–10 donor mice per cohort. Overall, this technique can be used in any laboratory (code provided in the Supplementary material).

In the best-performing XGBoost algorithm, in the test set, 7.9% of embryos from the young cohort were misclassified as aged. Indeed, there may have been embryos in the young cohort with poor morphokinetic performances that caused the algorithm to misclassify them. Even in young, pubertal control mice, a small proportion of embryos never reach the blastocyst stage (10–20%) [106, 107], presumably due to poor-quality oocytes. Thus, additional studies are needed to test whether the misclassified young embryos have poor reproductive potential as that of the aged group into which they were misclassified.

Third, this machine learning technique can be a non-invasive tool to describe embryo morphokinetics phenotypes while still preserving their cellular data. Morphokinetic patterns generated by AI are non-destructive compared to invasive interventions such as trophectoderm biopsy for preimplantation genetic testing for aneuploidy (PGT-A). Time lapse microscopy allows one to study and classify embryos during development and still allow molecular analysis on the same embryos, if desired. In other words, one can link this phenotype of the embryo to the molecular analysis result to study genotype–phenotype correlations. Examples of murine molecular analysis applications include embryo outgrowth assays and genetic screens, such as the Knockout Mouse Project [108], where morphokinetics can serve as a phenotype for preimplantation embryo screens.

The strengths of the study include the merger of AI with time-lapse microscopy to describe how early embryonic growth is changed due to the effect of maternal aging. We found that maternal aging is associated with accelerated cleavage stage and morula development and relative slowing of blastulation compared with their younger counterparts. This difference can be discerned using AI algorithms so that this aging phenotype may be predicted in future studies. Another strength of the study is the non-invasive approach of time-lapse microscopy. This allows one to gather comprehensive imaging data of each embryo without disturbing the external conditions such as temperature and carbon dioxide concentration. Thus, critical stages such as fading of the pronuclei are captured without physical interference.

A limitation of this study is that embryos were processed as a pooled cohort for each phenotype and thus how individual mice contributed to embryo development cannot be controlled. Further studies are needed to validate the findings. More studies will also be needed to test different mouse strains in different laboratory conditions and in greater numbers. With a larger dataset, other advanced AI techniques can be used such as time-lapse image classification [109]. Another limitation of this study is that arrested embryos were excluded from machine learning analysis due to a low number of embryos and lack of significant differences between the two experimental cohorts. Additional studies with greater numbers of arrested embryos could be incorporated into future studies.

Additionally, a limitation with our unsupervised machine learning is that default parameters were used in order to optimize faster training speed rather than optimized model in the context of high throughput of data. Therefore, additional classification methods should be tested in the future with parameters that optimize the model performance.

In summary, we show that maternal aging is associated with faster cleavage stage embryonic development in mouse and that AI can be used to predict this aging phenotype. These findings demonstrate that maternal aging alters embryo development using AI and morphokinetics and contributes to our understanding of reproductive aging with possible applications in embryo selection in IVF.

## Supplementary Material

Supplemental_File_1_R1_ioae056

Supplemental_File_2_ioae056

## Data Availability

The data underlying this article are available in the article and in its online supplementary material.
