# Supplementary material for: Machine learning in time-lapse imaging to differentiate embryos from young vs old mice
Source: Biol Reprod. 2024 Apr 30;110(6):1115–24. doi: 10.1093/biolre/ioae056 (PMC11180621; doi:10.1093/biolre/ioae056)
Supplement: Supplemental_File_1_R1_ioae056 [file supplemental_file_1_r1_ioae056.pptx]

## Slide 1
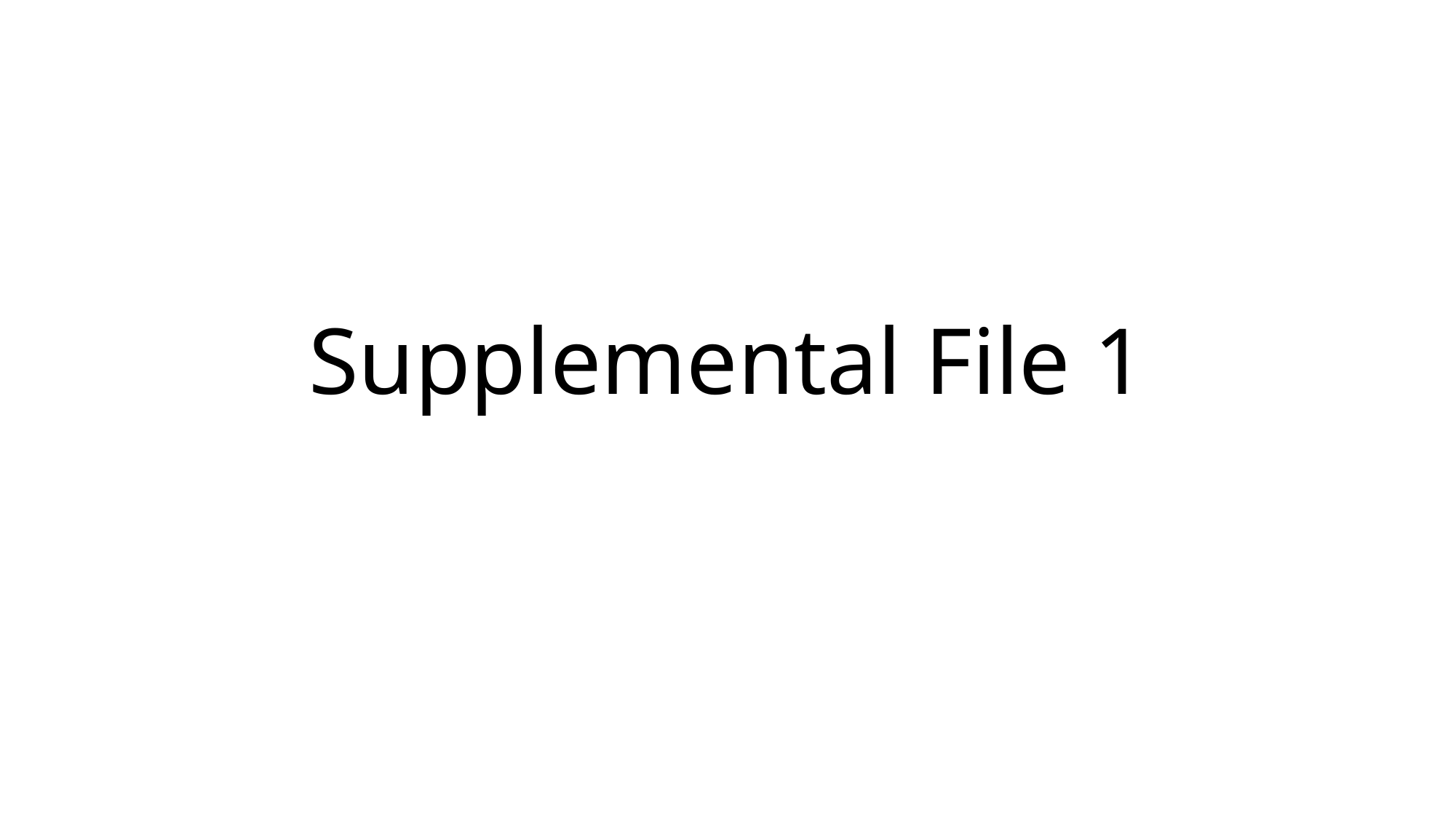

# Supplemental File 1

## Slide 2
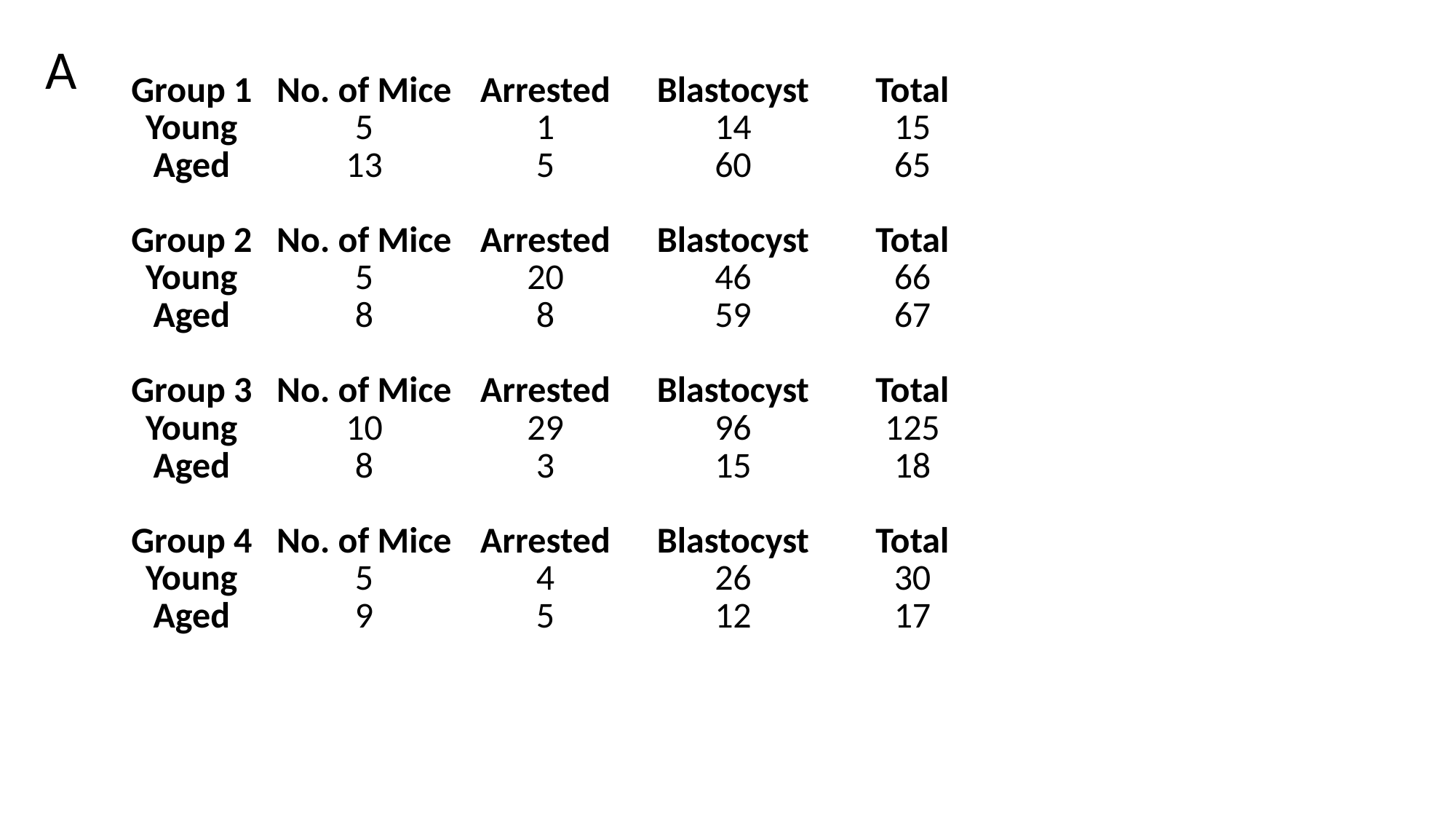

A
| Group 1 | No. of Mice | Arrested | Blastocyst | Total |
| --- | --- | --- | --- | --- |
| Young | 5 | 1 | 14 | 15 |
| Aged | 13 | 5 | 60 | 65 |
| | | | | |
| Group 2 | No. of Mice | Arrested | Blastocyst | Total |
| Young | 5 | 20 | 46 | 66 |
| Aged | 8 | 8 | 59 | 67 |
| | | | | |
| Group 3 | No. of Mice | Arrested | Blastocyst | Total |
| Young | 10 | 29 | 96 | 125 |
| Aged | 8 | 3 | 15 | 18 |
| | | | | |
| Group 4 | No. of Mice | Arrested | Blastocyst | Total |
| Young | 5 | 4 | 26 | 30 |
| Aged | 9 | 5 | 12 | 17 |

## Slide 3
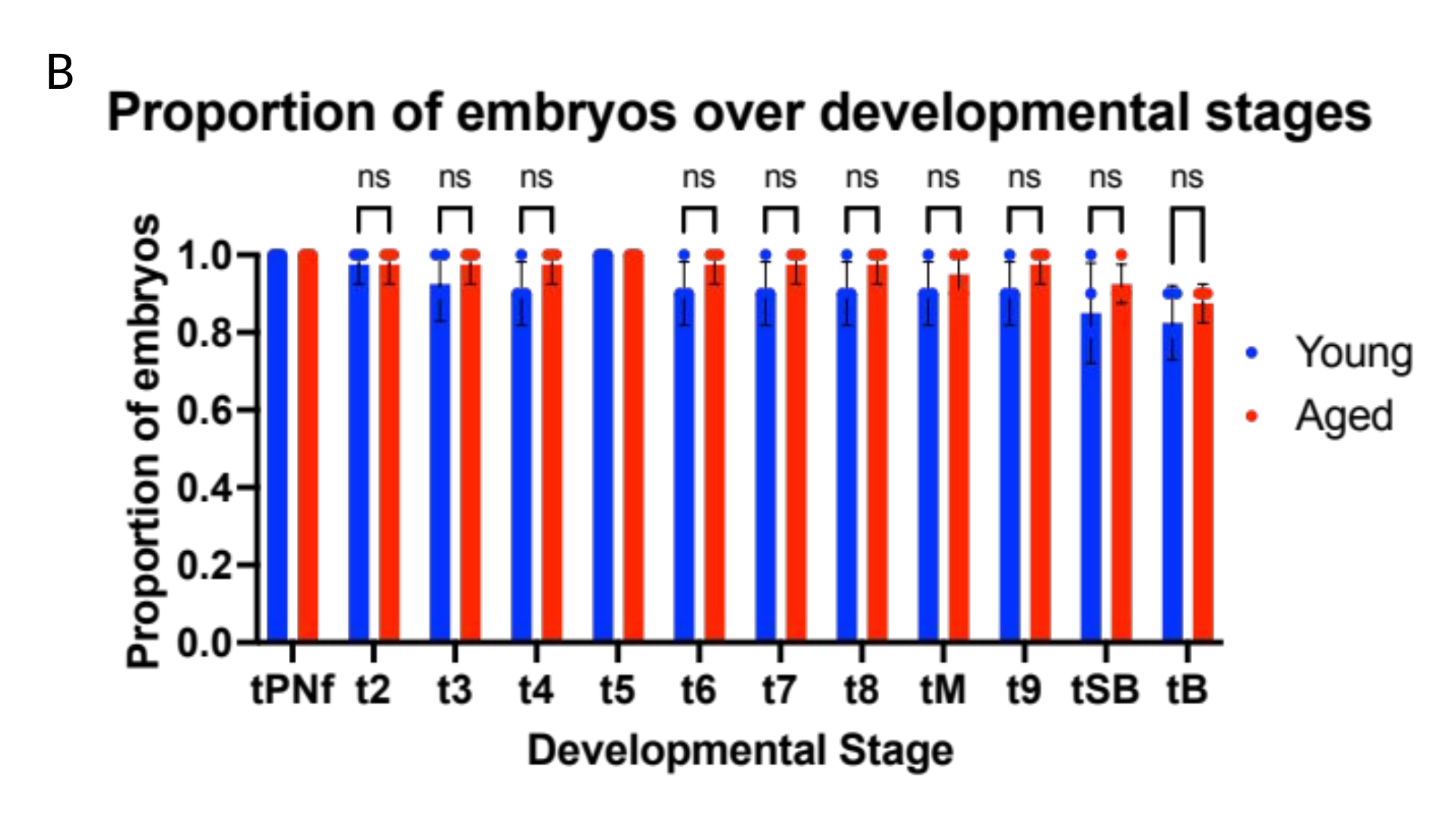

B

## Slide 4
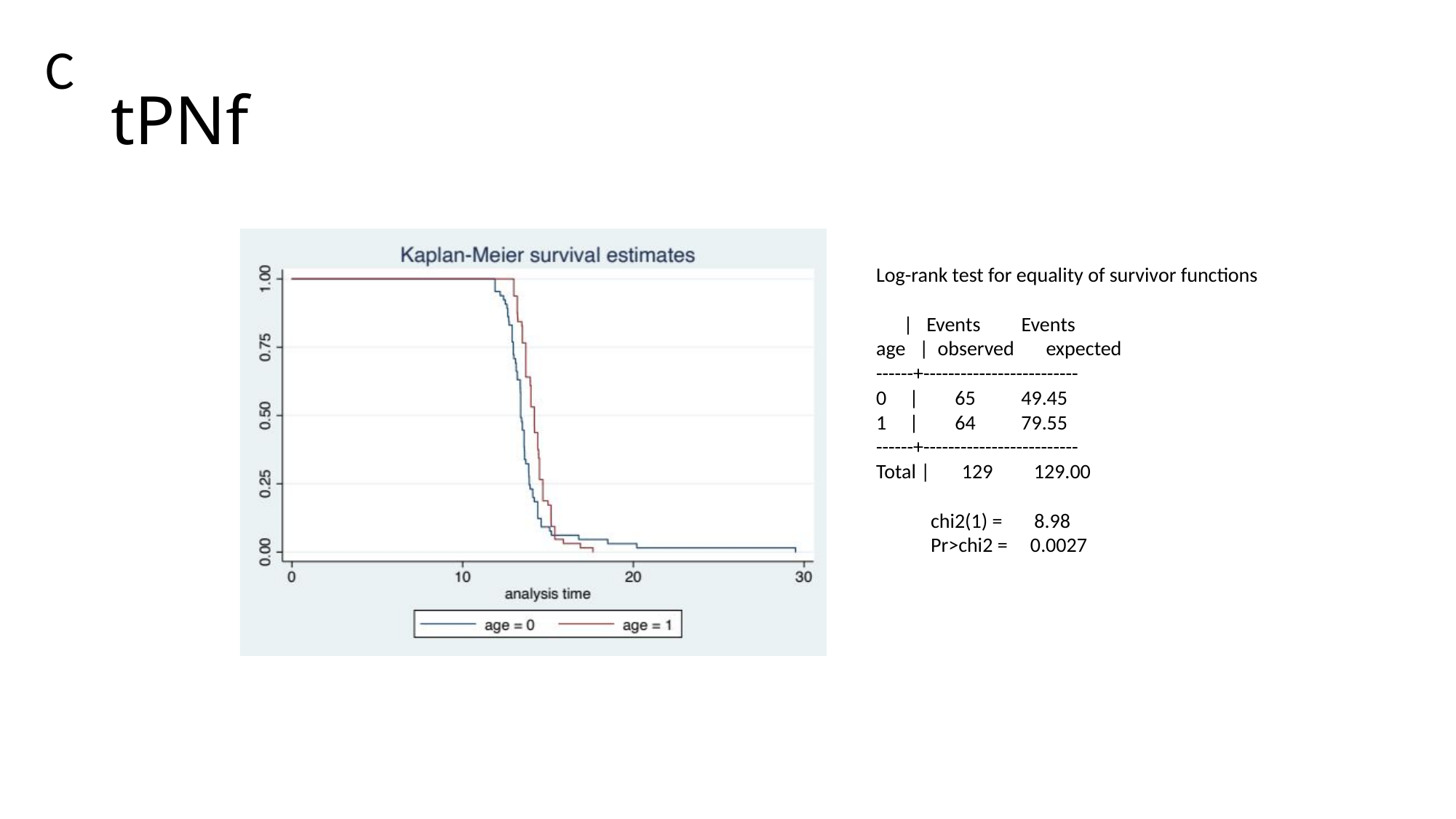

C
# tPNf
Log-rank test for equality of survivor functions
 | Events Events
age | observed expected
------+-------------------------
0 | 65 49.45
1 | 64 79.55
------+-------------------------
Total | 129 129.00
 chi2(1) = 8.98
 Pr>chi2 = 0.0027

## Slide 5
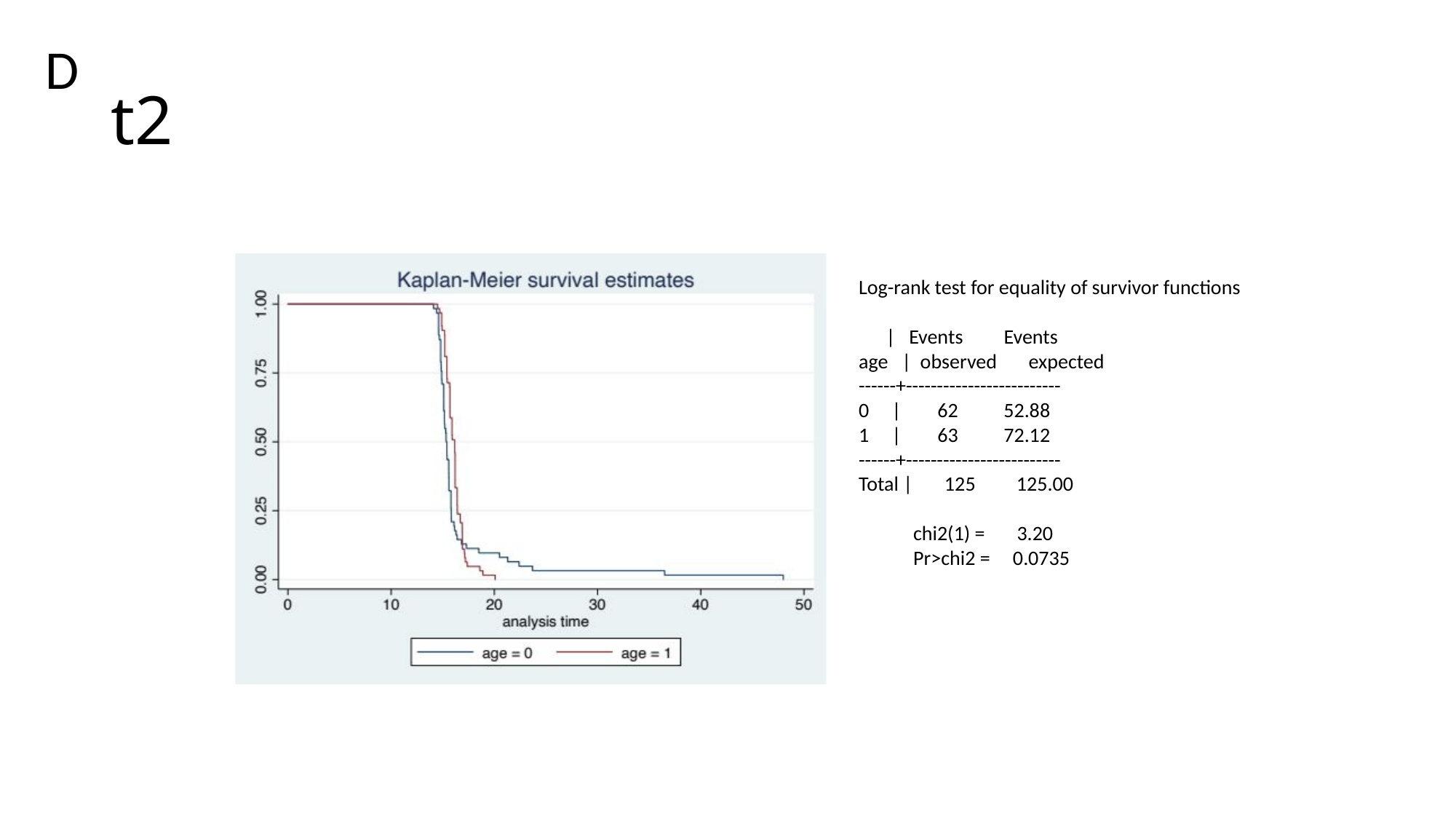

D
# t2
Log-rank test for equality of survivor functions
 | Events Events
age | observed expected
------+-------------------------
0 | 62 52.88
1 | 63 72.12
------+-------------------------
Total | 125 125.00
 chi2(1) = 3.20
 Pr>chi2 = 0.0735

## Slide 6
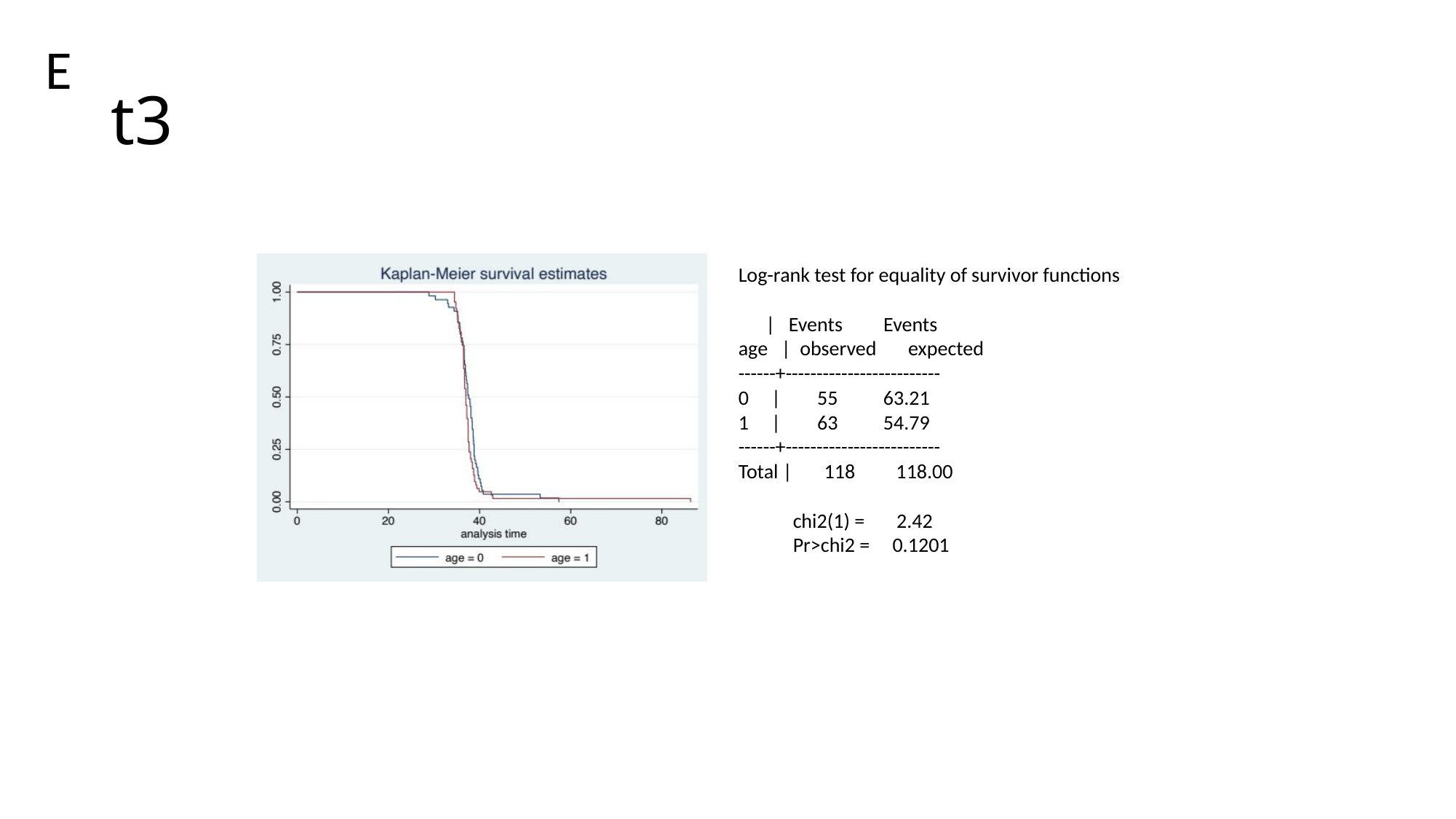

E
# t3
Log-rank test for equality of survivor functions
 | Events Events
age | observed expected
------+-------------------------
0 | 55 63.21
1 | 63 54.79
------+-------------------------
Total | 118 118.00
 chi2(1) = 2.42
 Pr>chi2 = 0.1201

## Slide 7
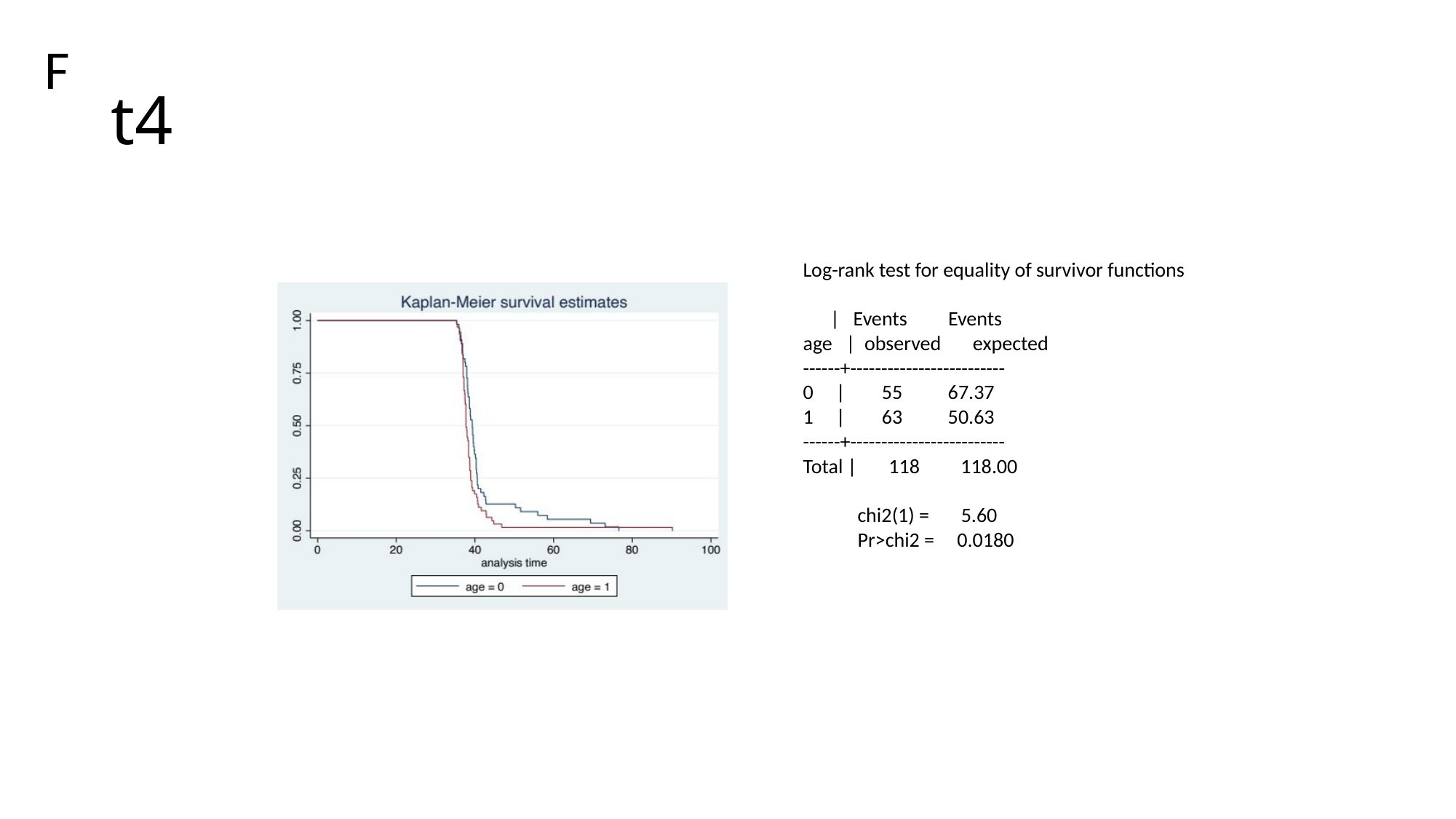

F
# t4
Log-rank test for equality of survivor functions
 | Events Events
age | observed expected
------+-------------------------
0 | 55 67.37
1 | 63 50.63
------+-------------------------
Total | 118 118.00
 chi2(1) = 5.60
 Pr>chi2 = 0.0180

## Slide 8
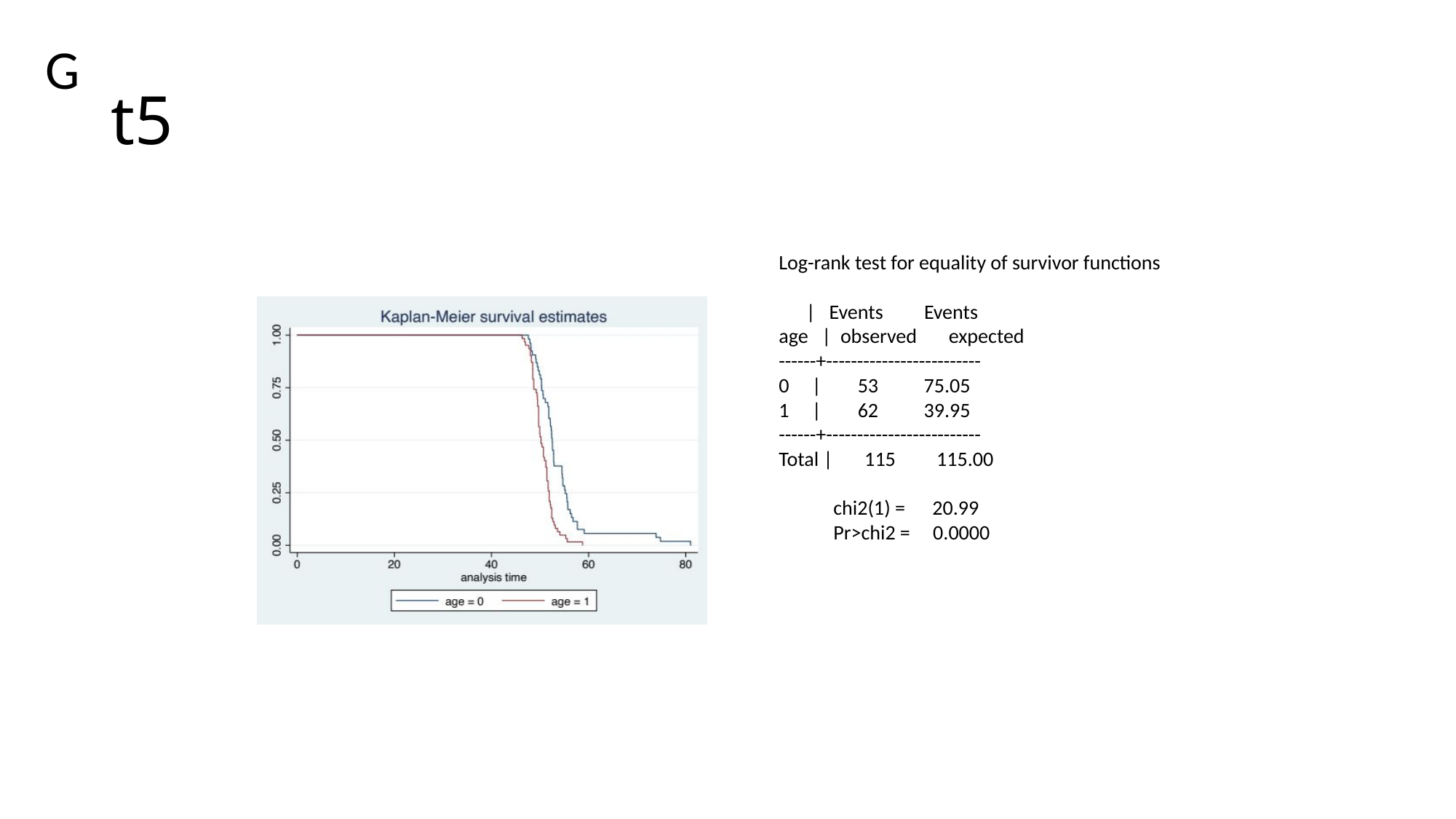

G
# t5
Log-rank test for equality of survivor functions
 | Events Events
age | observed expected
------+-------------------------
0 | 53 75.05
1 | 62 39.95
------+-------------------------
Total | 115 115.00
 chi2(1) = 20.99
 Pr>chi2 = 0.0000

## Slide 9
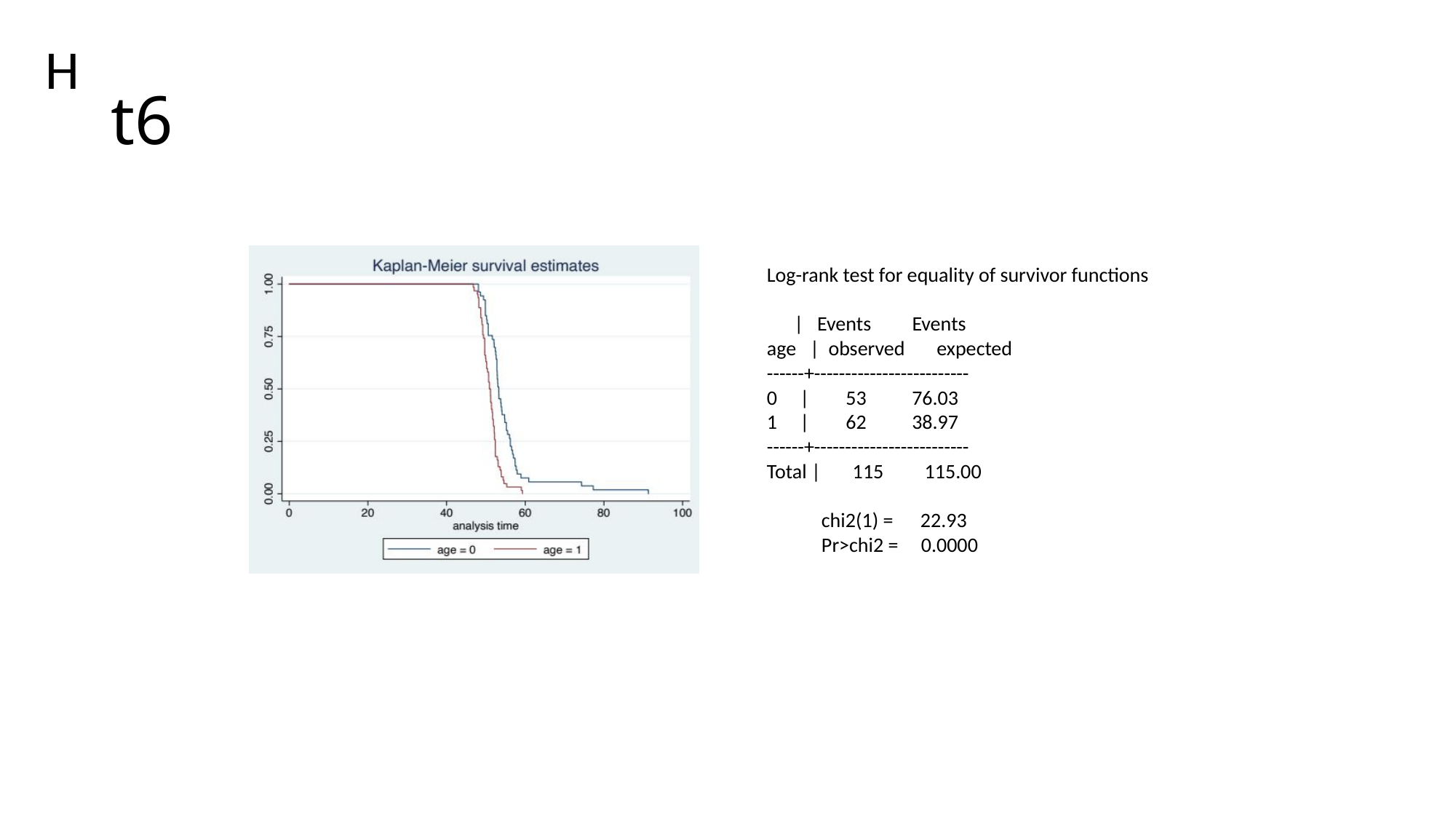

H
# t6
Log-rank test for equality of survivor functions
 | Events Events
age | observed expected
------+-------------------------
0 | 53 76.03
1 | 62 38.97
------+-------------------------
Total | 115 115.00
 chi2(1) = 22.93
 Pr>chi2 = 0.0000

## Slide 10
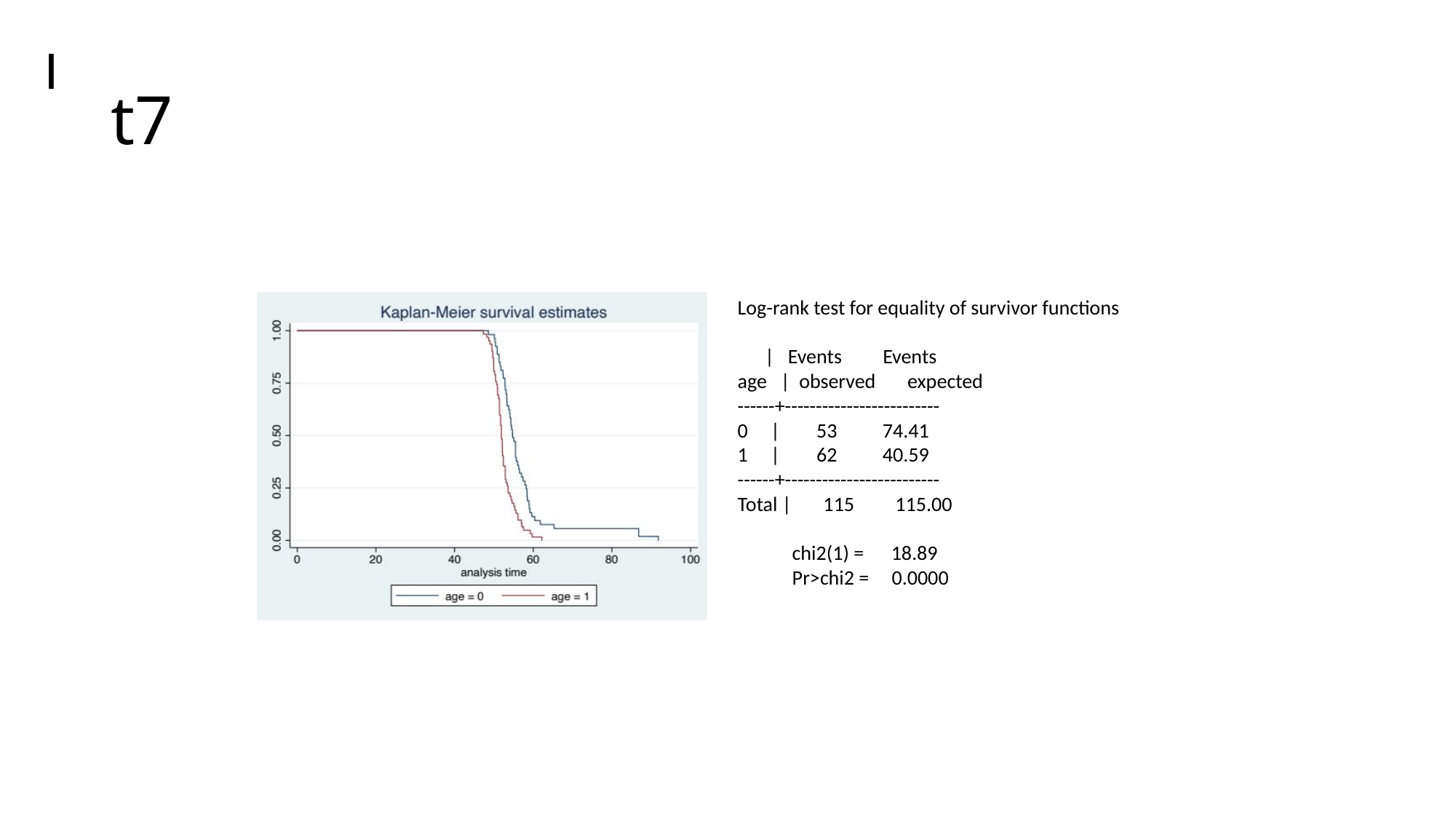

I
# t7
Log-rank test for equality of survivor functions
 | Events Events
age | observed expected
------+-------------------------
0 | 53 74.41
1 | 62 40.59
------+-------------------------
Total | 115 115.00
 chi2(1) = 18.89
 Pr>chi2 = 0.0000

## Slide 11
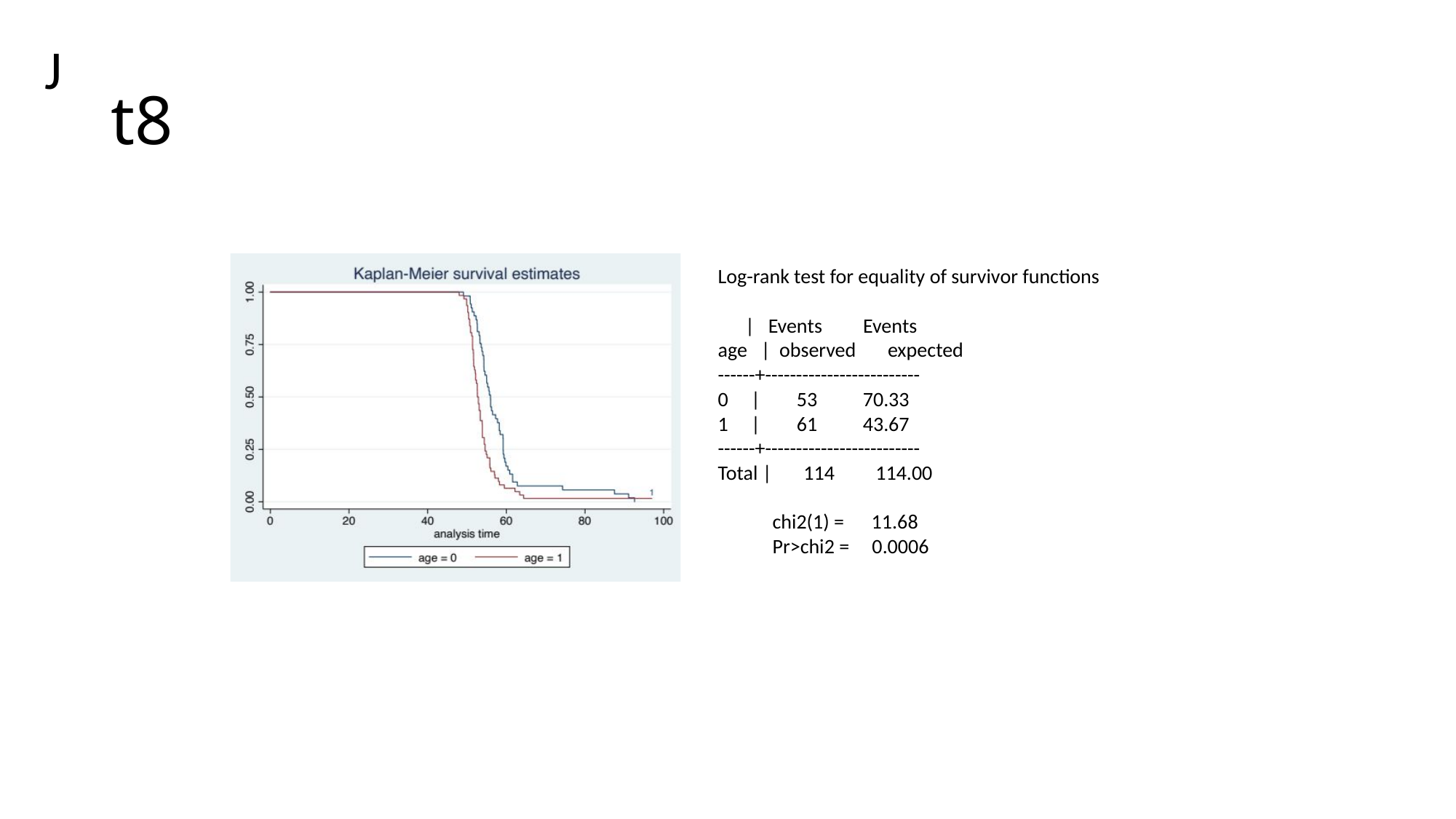

J
# t8
Log-rank test for equality of survivor functions
 | Events Events
age | observed expected
------+-------------------------
0 | 53 70.33
1 | 61 43.67
------+-------------------------
Total | 114 114.00
 chi2(1) = 11.68
 Pr>chi2 = 0.0006

## Slide 12
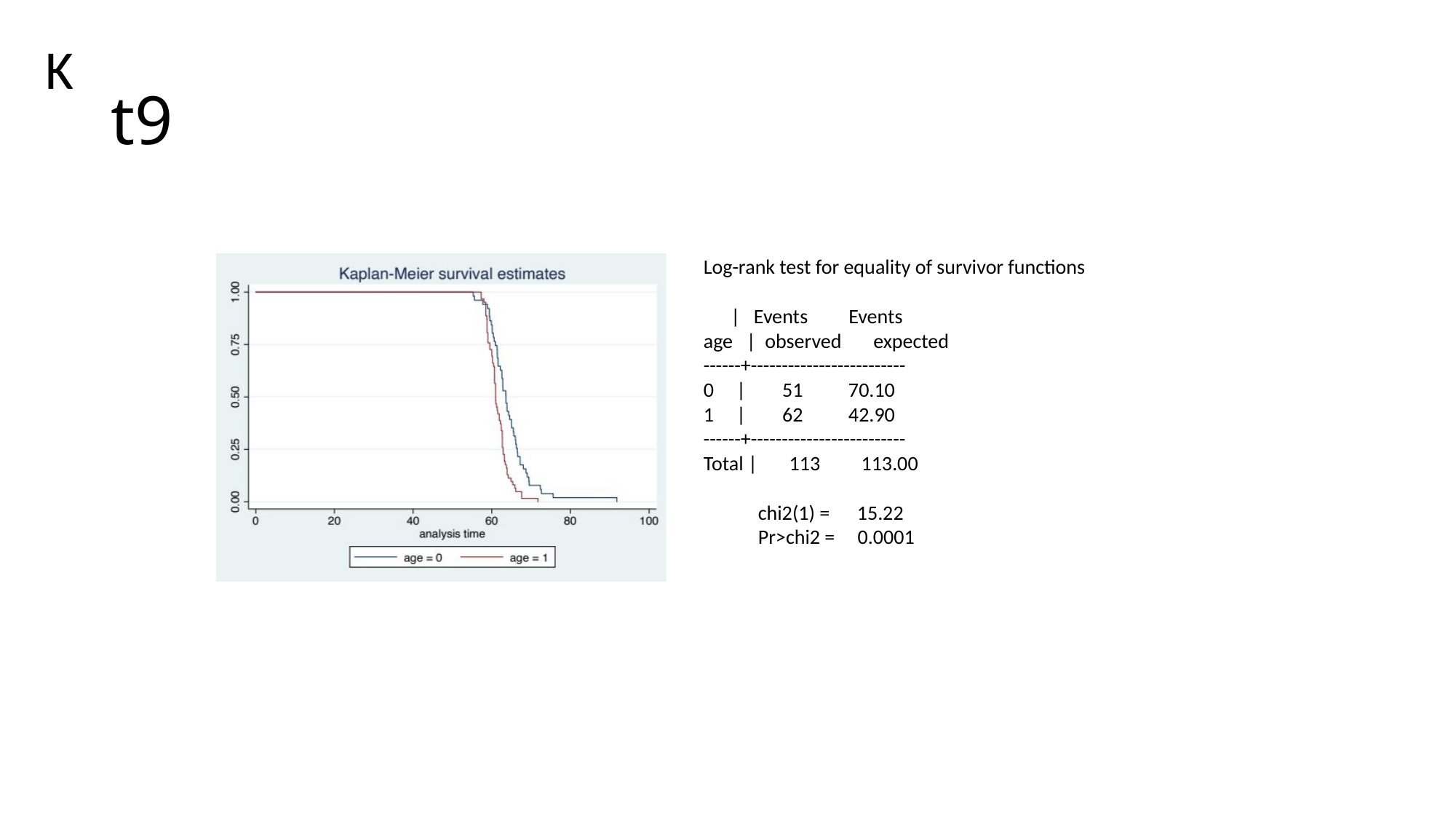

K
# t9
Log-rank test for equality of survivor functions
 | Events Events
age | observed expected
------+-------------------------
0 | 51 70.10
1 | 62 42.90
------+-------------------------
Total | 113 113.00
 chi2(1) = 15.22
 Pr>chi2 = 0.0001

## Slide 13
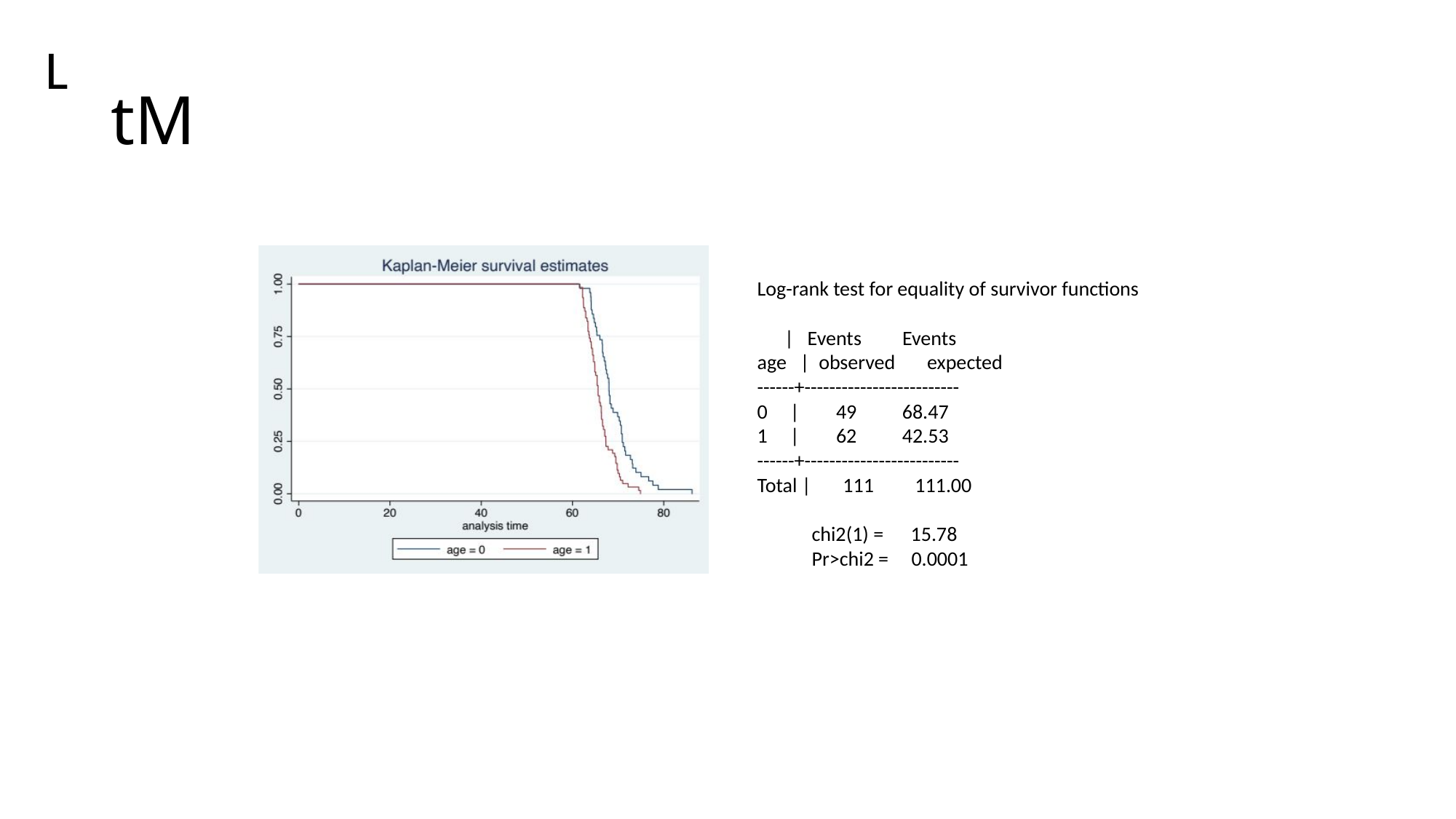

L
# tM
Log-rank test for equality of survivor functions
 | Events Events
age | observed expected
------+-------------------------
0 | 49 68.47
1 | 62 42.53
------+-------------------------
Total | 111 111.00
 chi2(1) = 15.78
 Pr>chi2 = 0.0001

## Slide 14
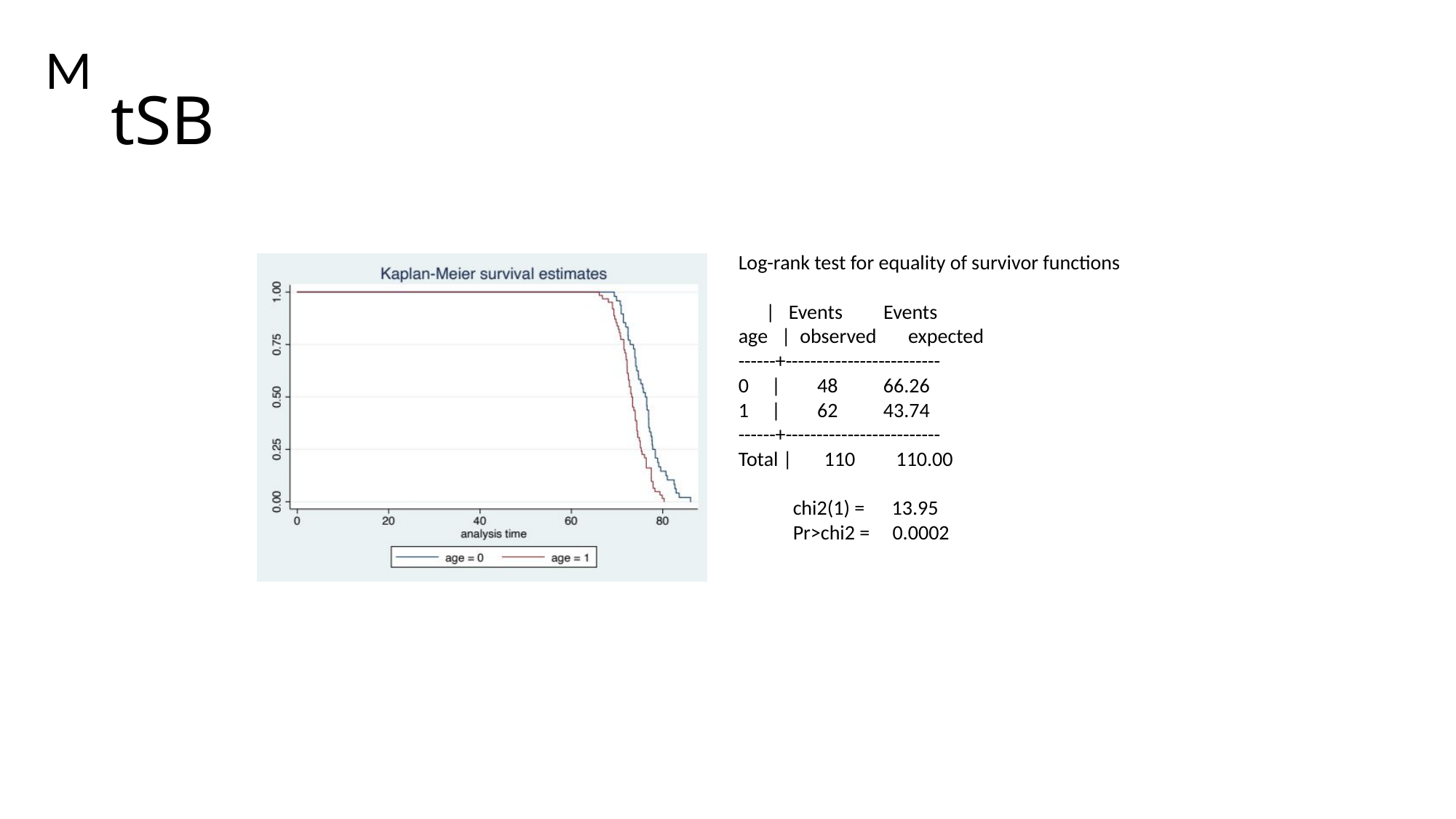

M
# tSB
Log-rank test for equality of survivor functions
 | Events Events
age | observed expected
------+-------------------------
0 | 48 66.26
1 | 62 43.74
------+-------------------------
Total | 110 110.00
 chi2(1) = 13.95
 Pr>chi2 = 0.0002

## Slide 15
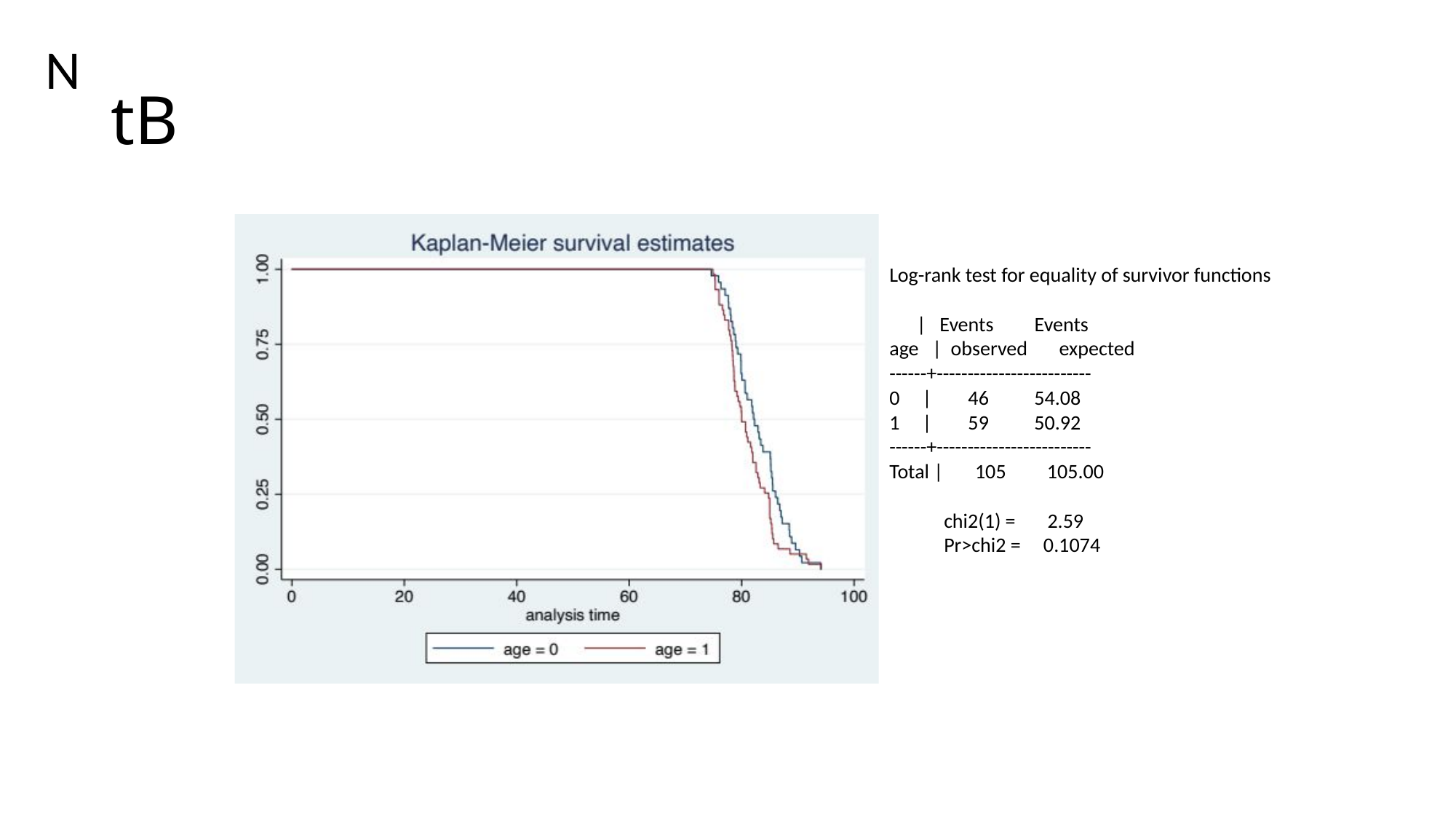

N
# tB
Log-rank test for equality of survivor functions
 | Events Events
age | observed expected
------+-------------------------
0 | 46 54.08
1 | 59 50.92
------+-------------------------
Total | 105 105.00
 chi2(1) = 2.59
 Pr>chi2 = 0.1074

## Slide 16
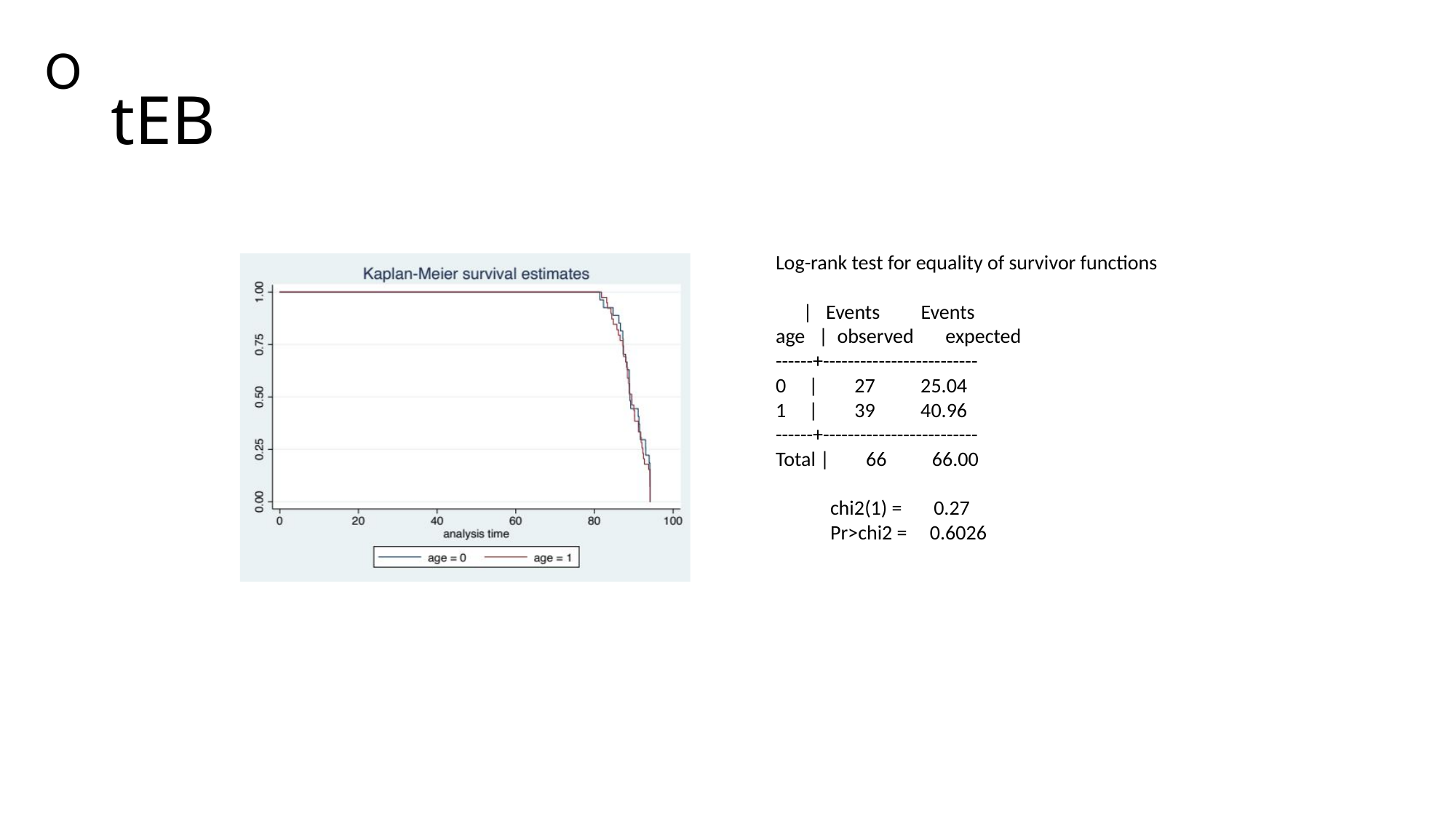

O
# tEB
Log-rank test for equality of survivor functions
 | Events Events
age | observed expected
------+-------------------------
0 | 27 25.04
1 | 39 40.96
------+-------------------------
Total | 66 66.00
 chi2(1) = 0.27
 Pr>chi2 = 0.6026

## Slide 17
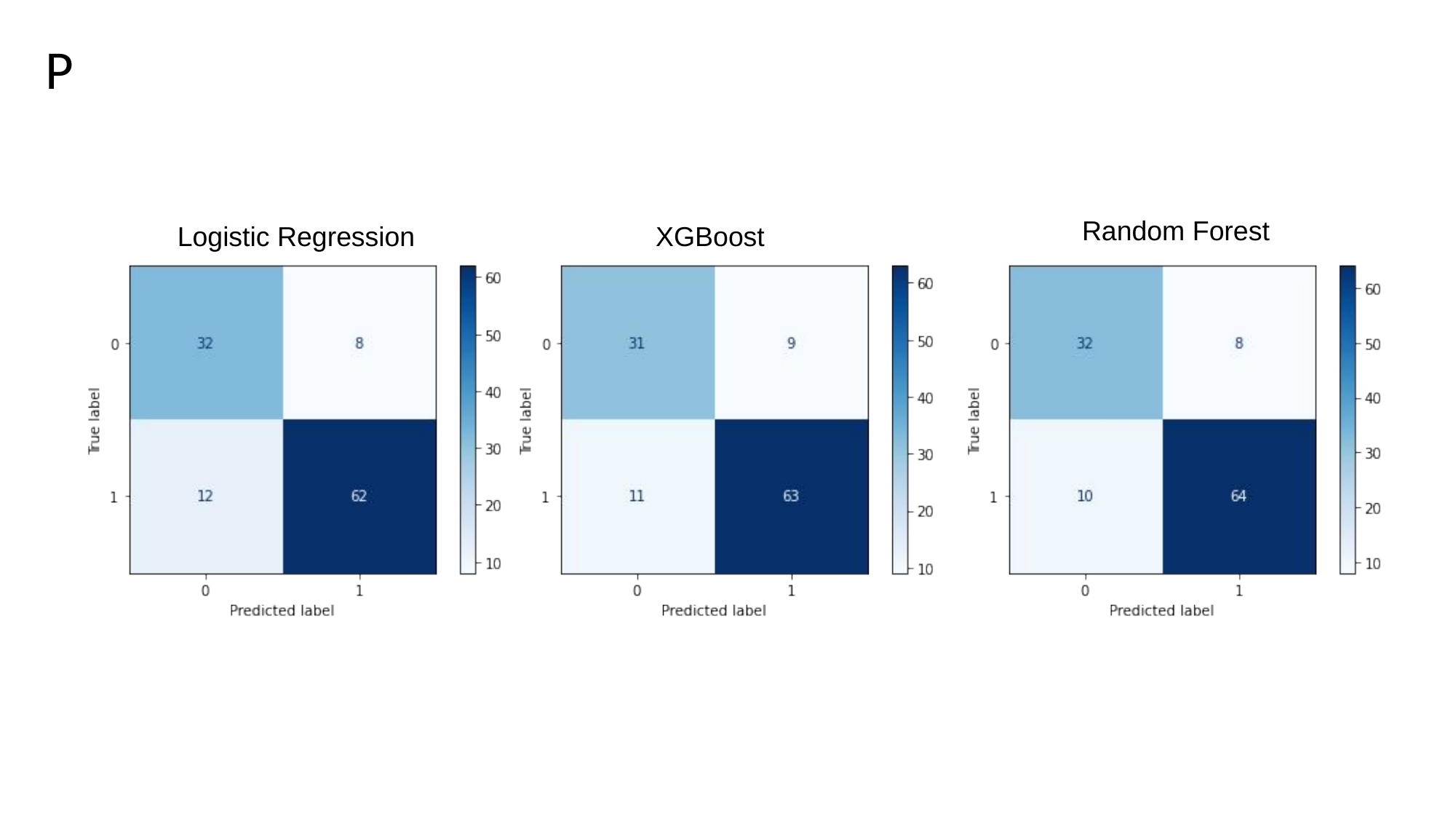

P
#
Random Forest
Logistic Regression
XGBoost

## Slide 18
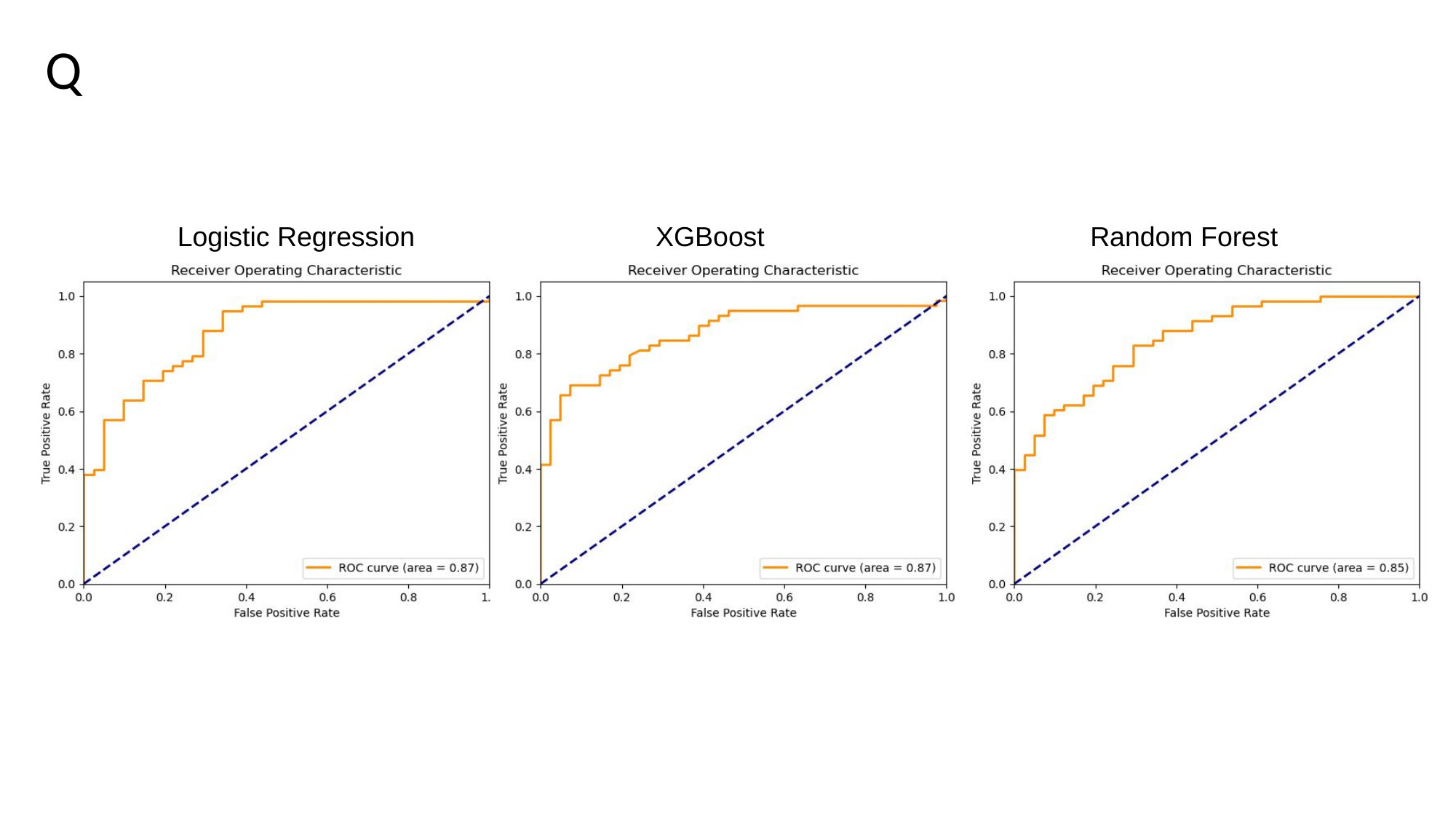

Q
Logistic Regression
XGBoost
Random Forest
